# An update on targeted gene repair in mammalian cells: methods and mechanisms

**DOI:** 10.1186/1423-0127-18-10

**Published:** 2011-02-02

**Authors:** Nanna M Jensen, Trine Dalsgaard, Maria Jakobsen, Roni R Nielsen, Charlotte B Sørensen, Lars Bolund, Thomas G Jensen

**Affiliations:** 1 Institute of Human Genetics, The Bartholin Building, University of Aarhus, 8000 Aarhus C, Denmark

## Abstract

Transfer of full-length genes including regulatory elements has been the preferred gene therapy strategy for clinical applications. However, with significant drawbacks emerging, targeted gene alteration (TGA) has recently become a promising alternative to this method. By means of TGA, endogenous DNA repair pathways of the cell are activated leading to specific genetic correction of single-base mutations in the genome. This strategy can be implemented using single-stranded oligodeoxyribonucleotides (ssODNs), small DNA fragments (SDFs), triplex-forming oligonucleotides (TFOs), adeno-associated virus vectors (AAVs) and zinc-finger nucleases (ZFNs). Despite difficulties in the use of TGA, including lack of knowledge on the repair mechanisms stimulated by the individual methods, the field holds great promise for the future. The objective of this review is to summarize and evaluate the different methods that exist within this particular area of human gene therapy research.

## Introduction

In the middle of the nineties, the field of targeted gene alteration (TGA) emerged as a possible method to correct diseases caused by single-base mutations [1,2]. Initially, the approach focused on stimulating the endogenous gene repair mechanisms using various single- or double-stranded oligonucleotides. These are complementary to part of the targeted gene except for one mismatched base specifically located at the site of the endogenous mutation. Upon cellular introduction these molecules will interact with the targeted gene sequence by different mechanisms. The mismatch is then recognized by components of the gene repair pathways, which subsequently can be stimulated to correct the mismatch by the use of the introduced targeting molecule [3-6].

Using TGA, mutated genes can be targeted and corrected without interfering with the endogenous promoter as well as enhancer/silencer elements and reading frames [7]. Such an impact has otherwise been seen with certain aspects of gene therapy introducing a complete gene sequence including all its associated elements [8,9]. Several methods have been developed in order to optimize and effectively implement the TGA strategy *in vitro *as well as *in vivo*. These methods all constitute different structures of targeting molecules, pathways of integration and gene repair pathways stimulated, resulting in variable success rates [4,10-12].

## Mammalian gene repair pathways

**Figure 1:**
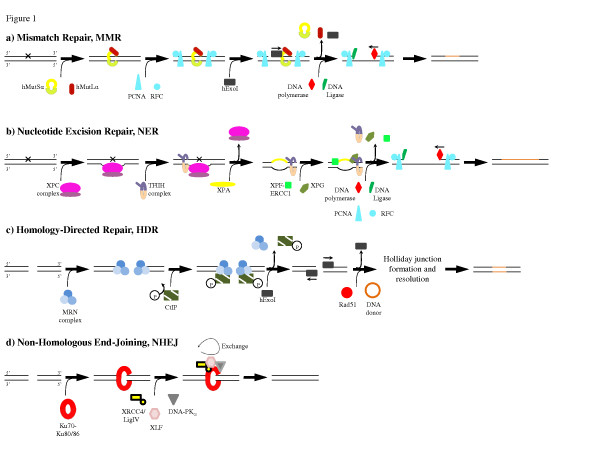
**Components involved in mammalian repair pathways**. A: In mismatch repair (MMR), hMutSα recognizes the DNA damage whereby hMutLα is recruited resulting in nicks on either side of the mismatch. Human exonuclease I (hExoI, 5'→3' activity) excises the mismatch and its flanking sequences after which DNA polymerase (3'→5' activity), along with PCNA and RFC, re-synthesizes a new DNA strand. B: In nucleotide excision repair (NER), the XPC complex recognizes the DNA damage causing the recruitment of the TFIIH complex, which unwinds the DNA to an open complex. XPA binds the damaged DNA strand after which endonucleases, XPG and XPF-ERCC1, excise the mismatch and DNA polymerase, with PCNA and RFC re-synthesizes the DNA strand. C: In homology-directed repair (HDR), the DSB is bound by the MRN complex recruiting CtIP and hExo, the latter of which excise nucleotides surrounding the break. Rad51 initiates homology search and when a homologous DNA donor is found, the DSB is repaired through Holliday junction formation and resolution. D: In non-homologous end-joining (NHEJ), the Ku complex recognizes the DSB leading to a simultaneous recruitment of DNA-PK_CS_, XRCC4:LigIV and XLF. The exchange of these factors drives the ligation of the non-homologous ends. Artemis nuclease, DNA polymerases μ and λ and other protein factors can be involved if the DNA ends are not directly compatible. See text for further details.

Mammalian cells utilize a variety of genetic repair pathways to ensure genomic stability of the genome. Understanding these pathways is essential for the further optimization of TGA [13-16]. A brief introduction to the pathways including their most central molecular factors is provided here (Figure 1). For detailed reviews see [17-23].

### Mismatch Repair (MMR)

The mismatch repair system (MMR) mainly corrects replication errors such as A-G and T-C mismatches [18]. It has been extensively studied both in prokaryotes and in mammalian cells, but for simplicity the following description will mainly focus on the mammalian homologues.

The recognition of mismatches in the mammalian MMR system (Figure 1A) is conducted by heterodimers of Msh (MutS homologue) proteins [24]. The Msh2:Msh6 heterodimer (hMutSα) recognizes base:base mismatches and small insertion/deletion loops, whereas the Msh2:Msh3 heterodimer (hMutSβ) recognizes 2-10 nucleotide insertion/deletion loops [25]. hMutSα-mediated mismatch recognition has been elaborately studied with less emphasis put on the mechanism conducted by hMutSβ. However, several similarities exist between the pathways [24]. hMutSα recognizes the mismatched base and binds to the damaged DNA strand, hereby recruiting hMutLα (hMlh1:hPms2 heterodimer) [19,24]. With the exchange of ADP for ATP, the hMutSα complex slides along the DNA strand causing hPms2-induced nicks on either side of the mismatch [17,19]. This enables entry of the exonuclease, hExoI, onto the 3'-end of the damaged strand, where it removes ~150 bases including the mismatch, after which replication protein A (RPA) is recruited to protect the newly exposed ssDNA [17]. DNA polymerase δ binds in association with its processivity factor proliferating cell nuclear antigen (PCNA) which is loaded onto the processed DNA by replication factor C (RFC) [24,25]. A new DNA strand is subsequently re-synthesized after which DNA ligase I joins the ends [17,19].

### Nucleotide Excision Repair (NER)

The nucleotide excision repair pathway (NER) (Figure 1B) primarily corrects bulky adducts and pyrimidine dimers caused by e.g. UV light [26]. Damage recognition is carried out by the XPC complex consisting of XPC, HR23B and Centrin-2, which binds to the non-damaged strand [20]. The TFIIH-complex, which is a heterodimer of 2 different helicases XPD (5'→3' activity) and XPB (3'→5' activity) attached to a cyclin-activated kinase (CAK) complex, is recruited and unwinds the double-stranded DNA surrounding the mutation [20,27,28]. An XPA-complex then binds to the damaged DNA strand followed by the arrival of an incision complex, consisting of the endonucleases XPG and XPF-ERCC1 [20]. This causes the excision of 25-30 nucleotides, including the damaged DNA, after which DNA polymerase δ (including PCNA) or DNA polymerase ε re-synthesize the DNA strand. Eventually, DNA ligase III re-joins the ends [20].

The recognition pathway involving the XPC-complex is named global genome repair (GGR) and corrects mismatches in the entire genome [27]. A transcription-coupled repair (TCR), which especially repairs actively transcribed genes, also exists. The damage recognition of this pathway involves the stalling of the RNA polymerase followed by recruitment of signaling molecules like Cockayne syndrome group A (CSA) and Cockayne syndrome group B (CSB) proteins [28]. Apart from the recognition step TCR functions as the GGR pathway [20].

### Base Excision Repair (BER)

Base excision repair (BER) corrects DNA mismatches caused by alkylation, deamination or oxidative damage [29]. Recently, it was shown that this pathway can be involved in one of the gene repair techniques (see single-stranded oligodeoxyribonucleotides) described in this review [30]. The DNA mismatch is recognized by DNA glycosylases which flip the damaged base out of the DNA helix and cleave it, creating an apurinic/apyrimidinic site (AP site) [29]. The DNA strand is subsequently cleaved by an AP endonuclease and an AP lyase creating a gap which is filled by DNA polymerase β and ligated by DNA ligase III [29]. A long-patch pathway of BER also exist where PCNA, DNA polymerase δ and DNA ligase I are among the proteins involved [29].

### Homology-Directed Repair (HDR) and Non-Homologous End-Joining (NHEJ)

Homology-directed repair (HDR) and non-homologous end-joining (NHEJ) are redundantly used to correct double-stranded breaks (DSBs) in the genome. Since these breaks are some of the most dangerous DNA damages occurring, these repair mechanisms play an important role in maintaining the integrity of the genome.

HDR repairs DSBs by the action of homologous recombination (HR) between homologous sequences using e.g. a sister chromatid as template (Figure 1C) [23]. After binding of the Mre11-Rad50-Nbs1 (MRN) complex, binding of CtIP is followed by human exonuclease I, hExoI, which trims the strands in a 5'-3'-directed manner. Replication protein A (RPA) is then recruited to protect the exposed ssDNA, before Rad51 initiates a homology search. When a homologous sequence has been detected, HR occurs through the formation and resolution of a Holliday junction [23].

NHEJ is the predominant mammalian DSB-repair pathway of the two, occurring at a ratio of approximately 1000:1 [31]. However, NHEJ re-ligates DNA ends without any use of homology, thus causing it to be highly error-prone [32]. The damage recognition factor of the NHEJ pathway is the heterodimeric protein complex Ku consisting of the two subunits, Ku70 and Ku86 (Figure 1D) [33]. Ku binds the break-induced DNA ends leading to the independent, but simultaneous, recruitment of DNA-PKcs, XRCC4:LigIV and XLF [21]. These latter factors are constantly exchanged with non-bound proteins, hereby driving the NHEJ reaction where the newly exposed DNA ends are ligated back together [21]. If the two DNA ends are not directly compatible for ligation several other protein factors, as e.g. Artemis nuclease, facilitates the end-joining reaction [22].

It is currently unknown how the cellular decision on using NHEJ or HDR is made. HDR seems to occur only in cells that are in the S/G_2 _cell cycle phase, whereas NHEJ does not seem to be phase-restricted, although repairing all damages happening in the G_1 _phase [21,34]. In either case, the 5'→3'-resection of the exposed DNA ends seem to play a pivotal role in the decision between the two pathways [34]. Blunt DNA ends are preferably corrected by NHEJ, whereas DNA ends corrected by HDR are usually trimmed by hExoI [23,34]. Furthermore, phosphorylation of the HDR-involved factor CtIP seems to commit the repair to the HDR pathway, but whether additional decisive factors exist is still debated [23].

## Targeted gene alteration

**Figure 2:**
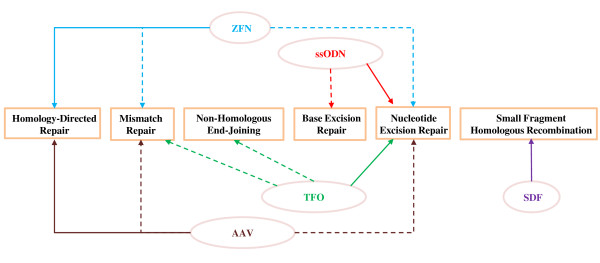
**Currently known connections between TGA-techniques and mammalian repair pathways**. Zinc finger nucleases (ZFNs, blue lines) function via homology-directed repair with the potential involvement of mismatch repair and nucleotide excision repair pathways. Single-stranded oligodeoxyribonucleotides (ssODNs, red lines) are believed to function via the nucleotide excision repair pathway with base excision repair potentially also playing a role. Triplex-forming oligonucleotides (TFOs, green lines) function via the nucleotide excision repair pathway with the possible participation of mismatch repair as well as non-homologous end-joining. Adeno-associated viruses (AAVs, brown lines) involve homology-directed repair and potentially also mismatch repair and nucleotide excision repair. Small DNA fragments (SDFs, purple line) are known to function via small fragment homologous recombination. See text for further details and references. Fully drawn lines refer to connections supported by experimental evidence from several groups whereas dotted lines refer to less substantiated links.

As previously mentioned, several different techniques can be used for altering mammalian genes through the activation of gene repair pathways. Overall, they can be divided into five categories, all of which will be discussed in the following. An overview of correlations between gene repair pathways and TGA techniques is illustrated in Figure 2 and a summary of important features of the TGA methods is supplied in table 1.

**Table 1:** Characteristics of TGA-mediating methods

**Method**:	ssODNs	SDF	TFO	AAV	ZFN
**Repair pathways involved**	NER, HDR? (MMR and NHEJ are suppressive)	SFHR	NER, NHEJ? MMR? HDR?	HDR, NHEJ	HDR, NHEJ

**Correction efficiency**^**a**^	0.1-5% (somatic cells) ~0.1% (ESCs)	0.2-20% (somatic cells) 0.025% (ESCs)	0.1-1.5% (somatic cells)	9.86%-65% (somatic cells) ~1% (ESCs and iPSCs)	~18-30% (somatic cells) 0.15-5% (iPSCs + ESCs)

**Advantages**	No integration of exogenous DNA, synthesis, stable, reproducible results	Reproducible results, potent episomal repair, artifacts can be circumvented	Synthesis, low toxicity, target specific, functional in hHPCs, stable target-complex formation	High efficiency and fidelity, effective in vivo delivery, broad cell type target field, low pathogenicity	High efficiency, known repair mechanism, normal cell cycle profiles, low background integrations, target silent genes

**Disadvantages**	Unknown repair mechanism, limited sequence size, PCR artifacts, genotoxicity, cell replication dependency	SFHR mechanism unknown, depend on HDR-like mechanism, synthesis (PCR)	Unknown repair mechanism, homopurine target restriction, G-C-rich sequences, weak DNA-binding, cellular death	Safety concerns, size limitation, integration of exogenous DNA, random integrations, cellular death	Synthesis, off-target cleavage, integration of exogenous DNA, multiple transductions

**Targeted disease genes**	Dystrophin α-D-glucosidase β-PDE TYR	CFTR DNA-PKcs Dystrophin β-globin SMN1	β-globin	COL1A1 COL1A2 FANCA Fah CFTR	CCR5 IL2Rγ CFTR HoxB13 TYR

**References**^**b**^	[4,9,12,14,41,46-49,51,52,54,62,116,117]	[4,8,35,39,40,63,64,118-121]	[16,66-69,80,84,122]	[4,11,31,54,85,88,90,92,93,123,124]	[6,10,12,13,102,104,114,125-127]

a) Note that the correction efficiencies might not be directly comparable due to differences in determination (e.g. efficiency vs. efficacy, factoring in targeting frequency, in vivo vs. in vitro conditions, etc.).

b) References used to construct table.

The polymerase chain reaction frequently forms the basis of assays involved in revealing effects of TGA-mediating methods and the reaction is furthermore used for production of small DNA fragments (SDFs) [35]. However, PCR is an error-prone reaction and even using highly accurate enzymes the DNA misincorporation frequency during a PCR reaction is high (~0.0035-0.02/bp) [36]. This may lead to uncertainty about whether unwanted mutations are introduced into the target gene when the desired mismatch is being corrected. Furthermore, the risk of PCR artifacts caused by priming of the corrective oligodeoxyribonucleotide (ODN) or SDF to the DNA can lead to false positives and produce an incorrect estimate of the correction efficiency [8,37]. Earlier this lead to criticism especially of SDF- and ODN-mediated gene targeting [37]. In order to avoid this, novel protocols have recently been developed. These include the use of analytical PCR-primers located outside the region of SDF/ODN-homology as well as gel purification of heat-denatured genomic target DNA [38-40]. Both of these methods contribute to an increased reliability of PCR-based assays. However, the lack of standardized, non-PCR-based assays of gene repair can make it difficult to compare the different methods directly [8,39]. Next generation sequencing methods will probably be used increasingly in order to document the repair frequencies and the integrity of the genome.

### Oligonucleotides

Single-stranded oligo-deoxyribonucleotides (ssODNs) have been used for TGA. The structure of ssODNs is simple and comprises a single-stranded DNA sequence complementary to the target site except for a single mismatched nucleotide located centrally in the molecule [3]. Phosphorothioate-conjugates as well as 2'-O-methylated uracil bases can be used to create modified ssODNs which exhibit high levels of stability through resistance to e.g. endogenous RNase H activity [41,42]. The invasion mechanism of these oligonucleotides is still unclear. However, several experimental results point to the involvement of DNA replication in the incorporation process with replication forks destabilizing the genomic nucleosome structure. Hereby, binding and subsequent incorporation of the ssODN at or near the replication fork - possibly as a "pseudo-Okazaki-fragment" in the lagging strand - is enabled [43,44]. This hypothesis is supported by evidence demonstrating that cell cycle arrest in the S-phase occurs in ssODN-treated cells. In these arrested cells cooperation between replication forks and the ssODN, including the search for homology, have sufficient time to occur [12]. However, the cell cycle arrest has been disputed and, if occurring, it seems to be temporary [30,45]. In either case, a cellular need for prolonged S-phase may pose problems in clinical applications with many in vivo targets undergoing only limited levels of replication and division [46].

Upon invasion, a 3-stranded heteroduplex is formed between the ssODN and the double-stranded target site [3,41]. Whether a correctional strand bias exists has been discussed and in several instances antisense ssODNs (i.e. ssODNs targeting the non-transcribed strand) has been giving the highest correction efficiencies [4,9,47-49]. This strand bias originally led to the conclusion that the transcription machinery and its accessory factors invoke a steric hindrance on the transcribed strand complicating the binding of ssODNs [50]. However, evidence show that the non-transcribed strand can be biased even when targeting transcriptionally silent genes [9]. This means that the transcription machinery is not solely responsible, if at all, for the strand bias seen with ssODNs and transcription-independent factors must be involved in the process [9,49]. In addition, studies show that two identical mutations at different locations of a target gene is repaired with opposing bias, indicating high target sequence dependency and in this case a low GC content in the flanking region favoring correction of the non-transcribed strand [51]. The specific repair mechanism underlying ssODN-mediated TGA is still disputed. However, a general consensus on the suppressive role of the MMR pathway has been established with several groups reporting a correction efficiency increase in Msh2-deficient cells [12,14,47,52,53]. The reason for this is not yet elucidated. However, Msh2 is known to suppress homeologous recombination, i.e. HR between nearly homologous sequences, potentially by functioning as an anti-recombinase - a phenomenon known as heteroduplex rejection [54,55]. On the basis of this, the Msh2 protein has been suggested to block ssODN-DNA heteroduplex formation at the replication forks because of the sequence divergence present here [14,54]. Likewise, cells lacking the mismatch repair endonuclease Pms2 also showed a higher level of ssODN-mediated TGA [46]. Recent results show that the cellular introduction of ssODNs leads to an increase in the amount of genomic DSBs [12,48]. This indicates a genotoxic effect of ssODNs but more notably that HDR could be involved in the TGA mechanism, despite the fact that ssODNs are complementary and not homologous to their target strands. Likewise, the presence of these DSBs could explain the aforementioned cell cycle arrest seen in ssODN-treated cells with HDR-mediated repair causing arresting phosphorylation of cell cycle checkpoint proteins [12,41]. Besides the involvement of MMR and HDR, the NER proteins, XPG and ERCC4 seems to be required to facilitate ssODN-mediated TGA, whereas components in the NHEJ pathway was found to inhibit the correction process [54,56]. The latter finding has been challenged however, with recent data showing that ssODNs compete for DSB-produced ends that would otherwise engage in NHEJ [57]. Furthermore, it was shown that single strand annealing (SSA) which is a repair pathway correcting DSBs occurring between repetitive DNA sequences is not involved in ssODN-mediated TGA, as otherwise described in yeast [57,58]. Recently, the involvement of another DNA repair pathway, known as base excision repair (BER), has also been implicated in ssODN-mediated TGA by the use of methyl-CpG-modified ssODNs [30]. These oligonucleotides are able to bind MBD4, a member of the BER pathway, and a gene correction efficiency increase of more than 10-fold compared to unmodified ssODNs was seen [30]. Methyl-CpG-modified ssODNs are restricted by the necessity of a guanine immediately 3' of the base targeted for repair [30].

However, the ability to correct single-base mutations without the incorporation of large pieces of exogenous DNA has made ssODN-mediated TGA thoroughly studied and employed in mammalian cells.

Chimeric RNA/DNA oligonucleotides (RDOs) are another type of oligonucleotides which have been investigated for TGA. Compared to ssODNs, the RDO structure is more complex with a hairpin structure comprising a DNA strand, homologous to the targeted strand, pairing with RNA-nucleotides flanking the mismatched base [3]. The all-DNA strand of the RDO has been shown to be the only active player in the TGA process [59]. To avoid degradation of the RNA-moieties by cellular nucleases these nucleotides are usually modified by 2'-O-methylation of the sugar units [60]. It is believed that upon target invasion a heteroduplex is formed causing cellular recognition of the newly formed mismatch and leading to nucleotide correction using the all-DNA RDO-strand as template [3]. RDOs are rarely used in gene correction studies today, primarily due to a lack of reproducibility of correction efficiencies [2,3,41,51,54,60-62].

### Small DNA-fragments

Small DNA-fragments (SDFs), also known as small homologous DNA fragments, can be used for TGA. The fragments usually comprise 400-1000 bp and are homologous to their DNA target sequence being able to concurrently modify up to 4 sequential basepairs in vitro as well as in vivo [40,63]. SDFs induce genetic modification by means of a homology-based mechanism known as small fragment homologous replacement (SFHR) [63,64]. The details of the SFHR mechanism are still unknown [64]. However, homologous pairing is believed to cause the endogenous DNA target sequence to be replaced by the exogenous SDF after the introduction of this fragment into the cell nucleus [63]. This replacement causes a genetic modification of the targeted mismatch. Surprisingly, the HDR repair pathway does not seem to be directly involved in the SFHR-mechanism. This is based on the finding of SDF-corrected cells expressing wildtype p53, which normally inhibits homologous recombination through binding of Rad51 and the MRN complex [64,65].

**Figure 3:**
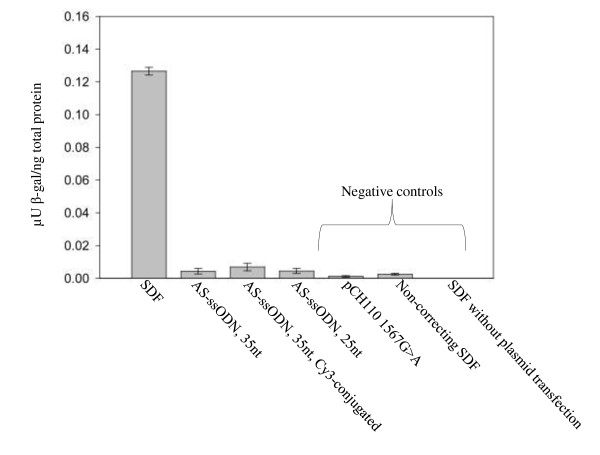
**Comparison between SDFs and ssODNs for correction of 1567G>A mutations in β-galactosidase genes**. CHO-K1 cells were co-transfected with the pCH110 1567G>A plasmid and correcting ssODNs (0.25 μM) or SDFs (7.5 nM) using 15 μg Lipofectamine (Invitrogen) [51]. Two days after transfection β-galactosidase enzyme activity was measured using a β-Galactosidase Enzyme Assay system (Promega) according to the manufacturer's protocol. ssODNs were designed to target the antisense strand (AS) of the β-galactosidase sequence in the region of the 1567G>A mutation. Two different lengths were employed: 25nt (AS-ssODN, 25nt) and 35nt (AS-ssODN, 35nt), both containing a centrally located cytosine in order to induce a mismatch with the targeted DNA. A Cy3-conjugated ssODN (AS-ssODN, 35nt, Cy3-conjugated) was included to test the effect of additional 5'-end protection. SDFs were synthesized using the pCH110 659G>A plasmid as template as previously described. The 480 bp SDF-molecule contained the mismatched base 270 bp from the 5'-end. As negative controls pCH110 1567G>A plasmid alone, a non-correcting SDF (constructed using the pCH110 1567G>A plasmid as template) and SDF without plasmid transfection were used.

SDFs can be created as either ds or ss DNA molecules - the latter by heat-denaturation of the double-stranded molecule [64]. Studies conducted using mammalian cells indicate no difference in correction efficiency between ss- and ds-SDFs [63,64]. However, a study carried out using *E. Coli *indicates a higher efficiency using ss-SDFs compared to ds-SDFs [35]. This may be due to circumvention of an SDF unpairing process, which in this study is suggested to be the rate-limiting step of the bacterial SFHR process [35]. Like several other TGA techniques including e.g. ODNs and TFOs (see below), SDFs have shown relatively high correction efficiencies within episomal target genes in vitro as well as in vivo [4,8]. SDF-mediated episomal gene repair has been reported in mouse embryonic stem cells and in human hematopoietic stem/progenitor cells [8,38,40]. However, the chromosomal correction efficiency obtained using the SFHR method is decreased compared to ssODNs, as opposed to the episomal repair efficiency [8]. The explanation for this disparity could be the increased mobility experienced by smaller molecules like ssODNs compared to larger molecules, possibly facilitating increased access to the nucleus [8]. In support of this notion we found that SDFs were superior to ssODNs in the correction of a 1567G>A mutation in episomal β-galactosidase genes (Figure 3). Furthermore, we used SDFs to correct mutations in β-galactosidase genes in vivo in mouse liver after hydrodynamic tail vein injection (unpublished results). SDFs have also been successfully employed for permanent ex vivo repair of the DNA-PKcs genes in a SCID mouse cell line [63].

In order to increase the correction efficiency of SDFs, ionizing radiation or treatment with Dox (doxorubicin), which inhibits topoisomerase II, has been employed [4,63]. The DSBs induced by these treatments are known to activate endogenous repair pathways relying on homologous recognition [4]. Besides Dox-treatment, cellular treatment with phleomycin which is a DNA-cleaving antibiotic able to cause S/G_2 _cell cycle shifts, results in a 5-fold correction efficiency increase on chromosomal targets [4]. This indicates SDF-mediated cell cycle phase dependency as well as an involvement of DNA replication in the SFHR mechanism, as reported for ssODN-mediated TGA.

An advantage of SDF-mediated gene modification is the reproducibility of results and no PCR artifacts occurring with the concentrations of SDFs used to produce high correction efficiencies (0.2-10%) [38,40]. However, lack of knowledge on the mechanism underlying SFHR and the error-prone PCR-based production method limits the use of this technique.

### Triplex-forming oligonucleotides (incl. peptide nucleic acids)

Triplex-forming oligonucleotides (TFOs) and peptide nucleic acids (PNAs) are single-stranded triplex-forming molecules exhibiting target sequence complementarity [66,67]. TFOs are short oligonucleotides (10-50 bp) consisting of RNA, DNA or synthetic derivatives (described later), which bind to the major groove of duplex DNA [67]. Hereby, the TFO functions as a 3^rd ^strand in a DNA-TFO-DNA triplex [67,68]. The specific binding is limited to homopurine tracts of the target sequence because the triplex is based on Hoogsteen bonds which are dependent on the available H-bond existing in purines [68,69].

Once bound to the targeted DNA, electrostatic repulsions originating between the TFO and DNA duplex are believed to trigger an, as yet, unknown series of DNA repair pathways [68,70]. The NER pathway has been shown important for this repair process, with TFO-mediated TGA not occurring in XPA- or CSB-depleted cells [70,71]. Furthermore XPC/Rad23B has been shown to recognize the TFO-induced triplex structure whereas XPD and XPF are believed to cleave the distorted DNA followed by strand re-synthesis by Polζ (polymerase ζ), which is involved in translesion bypass synthesis [68,72,73]. NER as well as MMR has furthermore been implicated in TFO-mediated TGA by the use of TFOs conjugated with the phototoxic mutagen psoralen. These modified TFOs induce TFO-directed psoralen interstrand crosslinks (Tdp-ICLs) which seem to be recognized by a multimeric complex consisting of either XPA-RPA (NER) and MutSβ (MMR) or XPC/Rad23B (NER) alone [16]. These results have lead to the proposal of TFO-mediated repair via an MMR-dependent error-free pathway as well as an NER-mediated error-prone pathway [16,74]. Furthermore, addition of TFOs along with a target-homologous DNA donor causes an increased gene correction efficiency leading to suggestions on the involvement of recombinatory repair pathways as well [75]. NHEJ is suggested to take over repair of Tdp-ICLs when NER factors are absent, whereas the necessity of Rad51 for TFO-induced recombination implicates HDR in TFO-mediated TGA [68,71]. In addition, a repair mechanism shift exist between longer (~30nt) and shorter (~10nt) TFOs with longer ones being repaired by NHEJ and shorter ones by NER [68,76].

Synthetic derivatives of nucleic acids used to create modified TFOs include methylene or ethylene bridged 2'-O, 4'-C's of the TFO backbone. These are known as bridged/locked nucleic acids (BNA/LNA) and ethylene nucleic acids (ENA), respectively, and are able to increase stability as well as correction efficiency under various physical conditions [77-79]. However, LNA-modified TFOs has yet to show a significant in vivo correction efficiency increase compared to unmodified TFOs [4]. This, in addition to a restriction to homopurine target sequences as well as weak DNA duplex binding at pH above 6, has made TFO-mediated TGA a subject for optimization [4,69,77,78].

PNAs provide a functional alternative to TFOs and are 12-18 nucleotides with a DNA backbone completely substituted by uncharged N-(2-aminoethyl)-glycine polyamides [80]. This modification highly increases the stability of the molecule through nuclease and protease resistance [80]. Furthermore, it enables a stable complex formation with the target DNA because of no electrostatic repulsions between the molecules [68]. This stability can be further enhanced by PNA-conjugation of the DNA intercalator molecule, 9-aminoacridine [81].

PNAs exist as three different variants: PNA oligomers, bis-PNAs and pseudo-complementary PNAs (pcPNAs) [66,82]. PNA oligomers can engage in either DNA-PNA-DNA triplexes like TFOs or in a PNA-DNA-PNA triplex invasion complex with the second DNA strand displaced as a P-loop [83]. Both of these complexes depend, at least partly, on Hoogsteen bonds causing a similar restriction to homopurine tracts as seen with TFOs. Likewise, bis-PNAs (2 PNA oligomers connected by a linker) induce PNA-DNA-PNA triplex invasion complexes [80]. These molecules have been shown to successfully correct a β-globin splice site mutation in primary hematopoietic progenitor cells [66]. However, target restriction to homopurine tracts is considered to be a major drawback of the triplexing method. Thus, double-duplex forming pcPNAs are the primary molecules used in PNA-mediated TGA today.

In pcPNAs, A and T nucleobases of the backbone have been replaced with pseudo-complementary 2,6-diaminopurine (D) and 2-thiouracil (U_s_) bases, respectively [84]. This incorporation sterically inhibits the otherwise stable PNA-PNA duplex formation and results in a double duplex invasion complex with the targeted DNA [69].  This type of invasion is solely dependent on Watson-Crick base pairing exempting pcPNAs from the homopurine target restriction [67]. Using N-(aminoethyl)-D-lysine entities the pcPNA backbone can be positively charged resulting in stable DNA duplex invasion complexes because of the electrostatic attraction between pcPNA and target [84]. The induced polarity furthermore enables invasion of G-C rich target sequences, which has otherwise been complicated by the lack of pseudo-complementary G-C nucleobases [84]. The modification has resulted in episomal correction frequencies of 0.65% [69]. However, the target sequence is still required to contain ≥50% A-T's in order to avoid PNA-PNA duplex formation [67]. Histone deacetylase (HDAC) inhibitor treatment following S-phase synchronization has furthermore lead to chromosomal correction efficiencies of 0.78% indicating a role for DNA replication in the mechanism of pcPNA-mediated TGA [69]. The uncertainties concerning the TFO-mediated repair mechanism apply for PNA-based technology as well, with the mechanism employed by these techniques believed to be similar, if not identical [69]. Since this mechanism has yet to be elucidated the use of pcPNAs for TGA is still not fully exploited.

### Adeno-associated virus vectors

Targeted gene alteration using vectors based on adeno-associated viruses (AAVs) has been studied for more than a decade. AAVs are icosahedral viruses consisting of a 4.7 kb single-stranded genome encoding rep- and cap-genes important for viral replication and capsid formation, respectively [85]. These genes are flanked by two inverted terminal repeats (ITRs, 145nt each), which are cis-acting elements necessary for viral transduction and functionality in TGA. The ITRs are the only original viral elements present in recombinant AAV vectors (rAAV), where rep- and cap-genes have been replaced by the homologous target-specific DNA before cellular introduction [4]. For production of the viral vectors the rep- and cap-genes are provided in trans.

After entry of the vector into the cell, target-specific homologous DNA is believed to activate and recruit HR-dependent repair factors, such as members of the MRN complex as well as Rad51 and Rad54 [86]. However, as described earlier, mammalian NHEJ is predominant compared to HDR for which reason homologous recombination is fairly undermined [31]. This is an obstacle that must be overcome since gene targeting is only seen when the DNA donor is enrolled in the HDR pathway. For this reason, several groups have studied transient knock-down of one or more protein factors known to be involved in the NHEJ pathway and this with success. By creating heterodimeric Ku70^+/- ^cells and using Ku70siRNA, it has been possible to increase gene targeting frequency at a chromosomal locus almost 9-fold [31]. Likewise, transient depletion of Ku70 and XRCC4, the latter being part of the XRCC4-LigIV complex responsible for NHEJ-mediated ligation, created an 11-fold increase in HDR-mediated repair [32]. However, a major restriction to the use of AAV vectors for TGA is the high ratio of random integrations (RI) to targeted HDR events seen in mammalian cells [5,87,88]. The transient knock-down of Ku70 did not appear to affect the RI frequency and with NHEJ believed to be the cause of RI, these results indicate the existence of a Ku70-independent NHEJ-pathway [31]. An alternative NHEJ-pathway (A-NHEJ) has indeed been reported, functioning in lymphoid cancers and being independent of Ku70 and XRCC4 as well as other important NHEJ-related factors [89]. However, the simultaneous depletion of Ku70 and XRCC4 caused a decrease of RI, suggesting that XRCC4 may simply be more pivotal than Ku70 in NHEJ-directed RIs [32].

As seen with SDFs [63], the introduction of DSBs as well as SSBs following the transduction process has demonstrated a significant increase in AAV-mediated correction efficiency reaching levels as high as 65% [88]. This increase supports the involvement of HDR and NHEJ in AAV-induced genetic correction. Furthermore, S-phase dependency seems important with the S/G_2_-arresting drug phleomycin leading to a 10-fold increase in the chromosomal correction efficiency of AAVs [4]. A direct correlation between intracellular AAV copy numbers and gene targeting frequency has been confirmed [11]. An advantage of AAV-based TGA is the success with which mesenchymal, hematopoietic and embryonic stem cells as well as induced pluripotent stem cells have been genetically targeted - with correction efficiencies ranging from 0.07-1% [90-93]. However, despite most groups only reaching stem cell efficiencies around 0.01-0.1%, the potential use of this technique to modify stem cells is revolutionary [5,11,91]. Based on high fidelity gene targeting, lack of pathogenicity and efficient in vivo delivery, AAV-mediated TGA shows great promise for the future.

### Zinc-finger nucleases

Zinc-finger nucleases (ZFNs) can be used for highly efficient TGA in mammalian episomal as well as chromosomal loci [13,94,95]. ZFNs are created by the fusion of 3-4 zinc-finger domains (ZFs), arranged in a ββα-fold coordinated by Zn^2+^, with the non-specific DNA-cleavage domain of the type IIS restriction enzyme, FokI [6,96,97]. Target specificity is determined by the amino-terminal end of the ZFs involved, and with the re-engineering of these domains, amino acid composition can be modified to induce highly specific ZFN-target binding [98]. The central feature of this technique is to induce DSBs in the DNA target which is done by dimerization of the FokI nuclease domains [99,100]. Therefore, ZFNs are produced in pairs with the FokI domains dimerizing at palindromic target sequences [10,99]. The ZFNs are designed to bind the targeted sequence in opposite directions recognizing a total of 18-24 bp [101]. This specificity ensures that only the targeted DNA sequence will be bound considering the size of the mammalian genome [102]. By supplying the ZFN pair to cells, genetic disruption is obtained by a FokI-facilitated DSB, which most likely is repaired by the NHEJ pathway resulting in permanent damage to the inflicted gene [103]. Conversely, if a DNA donor is simultaneously supplied to the ZFN-targeted cells genetic correction of the targeted sequence, through the activation of HDR, is achieved with HR of target and donor DNA [13].

The use of ZFNs for genetic correction has proven to be highly proficient with somatic gene correction efficiencies of ~18-30% being repeatedly reproduced and with human embryonic as well as hematopoietic stem cells being successfully targeted [6,13,95,104]. Surprisingly, the genetic correction of human CD34^+ ^hematopoietic progenitor cells has exhibited relatively low efficiencies (0.11%) compared to stem cells [13]. This divergence may be caused by poor growth as single cells, an ability necessary for specialized selection [95]. Furthermore, the lack of a single construct harboring the ZFN pair as well as the donor DNA might contribute to the low correction efficiencies due to complications concerning multiple transductions of progenitor cells [13,105]. Recent results show, however, that an optimal ratio between donor DNA and ZFNs is crucial to the gene correction efficiency in primary and adult fibroblasts as well as murine ES cells and primary astrocytes [106]. A donor DNA:ZFN ratio of at least 10:1 was shown necessary for optimal correction, indicating the importance of separate constructs harboring the ZFN pair and the donor DNA [106]. With the induction of a DSB *near *the site of mutation, the highest ZFN-mediated correctional efficiencies are reached - as seen with SDFs and AAVs [63,88,107]. In cases where designing a ZFN binding at the vicinity of the genomic mutation is impossible, genetic correction can be distantly stimulated [102]. ZFNs inducing HR at a distance of 400 bp has been successfully employed - however, at a decreased recombination frequency [102].

Promising results have been obtained using an integrase-defective lentiviral vector (IDLV) delivery method for ZFNs with somatic correction efficiencies reaching 29% [13]. However, these results were questioned due to the lack of southern blot analysis eliminating potential RIs of the donor DNA as well as documenting the actual HR process [95,108,109]. Random integration of IDLVs in the human genome has likewise been detected, posing a serious risk of unintended genetic modification [13,110]. Likewise, the extent of ZFN-mediated genotoxicity is still unresolved. A decreased phosphorylation of the mammalian damage sensor protein H2AX in ZFN-corrected cells compared to ssODN-treated cells indicates a tolerable level or complete lack of ZFN-induced genomic damage [12]. These results are further exciting due to the evidence of no misintegration of the donor DNA plasmid as well as no gross chromosomal rearrangements following ZFN-mediated genetic correction [6]. However, this conclusion could be challenged by reports of high frequencies of off-target cleavages by the ZFN pair, most likely caused by homodimerization of the individual ZFN-FokI domains [99,102]. The problem may be solved by the addition of positive or negative charges to the individual ZFN during the construction of these, causing electrostatic repulsion among identical ZFNs [10,96,99]. Experiments performed using this type of charged ZFNs shows a 40-fold reduction in off-target cleavages whereas arresting the targeted cells in the G_2_/M phase increased the HR:RI ratio almost 6-fold [99,111]. Shortening the half-lives of ZFN molecules by adding an N-terminal arginine resulted in reduced genotoxicity without decreasing the targeting efficiency [112]. Other factors affecting ZFN-mediated genotoxicity are the number of ZFs used with 4 being less toxic than 3, and the length of the ZF-FokI peptide linker with 4 amino acids being superior to 6 [102,113].

The construction of the complex ZFN molecules has earlier posed a major drawback to the use of these for genetic modification [114]. Originally, the ZFNs were constructed by the use of a modular assembly-method which encompasses the fusion of individual ZFs with established DNA-binding specificities [115]. Despite the relative ease with which this is performed, the efficiency of creating a functional ZFN pair is extremely low (<6%) [114,115]. However, with the construction of the publically available platform OPEN (Oligomerized Pool ENgineering) the design of ZFNs has become easier as well as safer [114,115]. Currently, the development of the ZFN-based technique is influenced by extensive patenting complicating the progression of the technique [94]. But with initiatives like the Zinc Finger Consortium providing public access to information concerning ZFN construction as well as expiration of predominant patents, this area is under constant development [114].

## Conclusion

The ability to correct genomic mutations and repairing cellular defects has been the centre of extensive research for several decades. Successful studies have been made with the transfer of full-length genes, but a constantly emerging problem concerns the regulatory elements of the gene of interest. However, this problem has been circumvented with the emerging of targeted gene alteration, which is based on the stimulation of endogenous cellular repair mechanisms, i.e. no interfering with any regulatory elements whatsoever. Targeted gene alteration functions via the addition of a variety of oligonucleotides including single-stranded oligonucleotides, small DNA fragments, pseudo-complementary peptide nucleic acids, adeno-associated virus vectors and zinc-finger nucleases. The former techniques rely on target-complementary oligonucleotides constructed by the use of standardized or synthetic nucleic acids. They have mainly received attention due to the ease and low cost with which they are synthesized as well as the stability of the molecules. However, gene correction efficiencies have generally been low in somatic cells (0.1-20%) and extremely low in various stem cells (~0.1%). Furthermore, the lack of knowledge concerning the different genetic repair mechanisms stimulated by one of these methods complicates optimization of the techniques. Conversely, the latter techniques are based on target-homology and stimulate genetic repair efficiency by the activation of the homology-based repair mechanism, HDR. However, the error-prone NHEJ is an unwanted side effect of this stimulation for which reason focus has been put on the cellular shut-down of this pathway in order for HDR to dominate. This has proven to be successful and AAVs and ZFNs obtain gene correction efficiencies as high as 65% in somatic cells and 5% in stem and progenitor cells. Despite their difficulty in synthesis and potential safety concerns regarding viral pathogenicity these techniques appear very promising for future studies on targeted gene alteration.

In this article, we have reviewed the methods currently used in targeted gene repair and the underlying mechanisms. Although clinical gene therapy has been undergoing extensive progress within the last two decades, gene repair for clinical applications is still in its infancy. The level of chromosomal gene correction efficiencies has, until recently, been too low for clinical translation. The key to enhanced gene correction efficiency currently lies with an in-depth understanding of mammalian gene repair mechanisms involved in the different TGA techniques. Furthermore, development of robust assays to compare the efficiencies is necessary.

Efficient gene correction in progenitor cells is required to permanently correct heritable genetic diseases. The rapid evolution of efficient methods for generating pluripotent stem cells and improved ex vivo culture methods will certainly improve our possibilities. Furthermore, the development of Zinc-finger nucleases and the use of adeno-associated virus vectors for gene repair have made it possible to induce efficient gene correction both in vitro and in vivo. This certainly has shortened the distance to clinical trials. However, safety issues concerning ZFN-mediated genotoxicity, off-target cleavages, AAV-based viral concerns and random integrations still remain to be solved, but high throughput sequencing methods to check the outcome of the repair efforts are already available. Thus, we are not in doubt that clinical applications of gene repair techniques have a great future - initially for monogenic disorders.

## Abbreviations

AAV: adeno-associated virus; BNA: bridged nucleic acid; CAK: cyclin-activated kinase; CSA: Cockayne syndrome group A; CSB: Cockayne syndrome group B; ds: double-stranded; DSB: double-stranded break; ENA: ethylene nucleic acid; HDAC: histone deacetylase; HR: homologous recombination; ITR: inverted terminal repeat; LNA: locked nucleic acid; MDB4: methyl-CpG binding domain protein 4; MRN: Mre11-Rad50-Nbs1 complex; PCNA: proliferating cell nuclear antigen; Pc-PNA: pseudo-complementary peptide nucleic acid; PNA: peptide nucleic acid; RDO: chimeric RNA/DNA oligonucleotide; RFC: replication factor C; RI: random integrations; RPA: replication protein A; SDF: small DNA fragment; ss: single-stranded; SSB: single-stranded break; ssODN: single-stranded oligo-deoxyribonucleotide; Tdp-ICL: TFO-directed psoralen interstrand crosslinks; TFO: triplex-forming oligonucleotide; TFIIH: transcription factor II H; TGA: targeted gene alteration; XPA/XPB/XPC/XPD/XPF/XPG: xeroderma pigmentosum A, B, C, D, F and G, respectively; ZFN: zinc-finger nuclease.

## Competing interests

The authors declare that they have no competing interests.

## Authors' contributions

The manuscript was prepared by NMJ and TGJ. All authors read and approved the final manuscript.
